# Cancer mortality in Yukon 1999–2013: elevated mortality rates and a unique cancer profile

**DOI:** 10.1080/22423982.2017.1324231

**Published:** 2017-06-09

**Authors:** Jonathan Simkin, Ryan Woods, Catherine Elliott

**Affiliations:** ^a^ School of Population and Public Health, Faculty of Medicine, University of British Columbia, Vancouver, British Columbia, Canada; ^b^ Office of the Chief Medical Officer of Health, Department of Health and Social Services, Government of Yukon, Whitehorse, Yukon, Canada; ^c^ Cancer Control Research, British Columbia Cancer Agency, Vancouver, British Columbia, Canada

**Keywords:** Cancer, epidemiology, prevention & control, mortality, rural health, circumpolar health, Yukon Territory, public health, population health

## Abstract

**Background**: Although cancer is the leading cause of death in Canada, cancer in the North has been incompletely described.

**Objective**: To determine cancer mortality rates in the Yukon Territory, compare them with Canadian rates, and identify major causes of cancer mortality.

**Design**: The Yukon Vital Statistics Registry provided all cancer deaths for Yukon residents between 1999-2013. Age-standardised mortality rates (ASMRs) were calculated using direct standardisation and compared with Canadian rates. Standardised mortality ratios (SMRs) were calculated using indirect standardisation relative to age-specific rates from Canada, British Columbia (BC), and three sub-provincial BC administrative health regions : Interior Health (IH), Northern Health (NH) and Vancouver Coastal Health (VCH). Trends in smoothed ASMRs were examined with graphical methods.

**Results**: Yukon’s all-cancer ASMRs were elevated compared with national and provincial rates for the entire period. Disparities were greatest compared with the urban VCH: prostate (SMR_VCH_=246.3, 95% CI 140.9–351.6), female lung (SMR_VCH_=221.2, 95% CI 154.3–288.1), female breast (SMR_VCH_=169.0 95% CI, 101.4–236.7), and total colorectal (SMR_VCH_=149.3, 95% CI 101.8–196.8) cancers were significantly elevated. Total stomach cancer mortality was significantly elevated compared with all comparators.

**Conclusions**: Yukon cancer mortality rates were elevated compared with national, provincial, urban, and southern-rural jurisdictions. More research is required to elucidate these differences.

## Introduction

In Canada, cancer mortality rates are declining overall. However, cancer remains the leading cause of premature death, with an estimated 78,000 Canadians dying of cancer in 2015 [1]. In 2008, cancer accounted for 40% of potential years of life lost and was the costliest disease in terms of lost productivity due to death, estimated at $166 million [1]. Despite progress in cancer prevention and control, the cancer burden is not evenly distributed across Canada [1–4]. Cancer prevention and control strategies should be underpinned by knowledge of local cancer mortality patterns.

Disparities in Canadian cancer care and outcomes have been reported between rural and urban populations [2–7]. For example, in British Columbia (BC), population-based studies have found lower utilisation of radiotherapy among lung and breast cancer patients living in rural areas with limited access [5,6]. Limited access to radiotherapy may influence treatment options. For example, rural breast cancer patients have been found to undergo mastectomy as opposed to breast-conserving surgery more often than patients within urban areas [2,5,6]. Previous reports have also found that cancer mortality is worse among lung, prostate, and colorectal cancer patients residing in rural versus urban Canada [2,7]. Similar observations have been reported in the United States and Australia [7–9].

Rural–urban disparities have been attributed to elevated risk factors such as smoking and disadvantages in social determinants of health, such as socioeconomic status and education, as well as physical and cultural barriers to accessing cancer care and prevention services [2–4,8,9]. Further, previous studies have noted that Canadian rural patients have poorer access to end-of-life services compared with their urban counterparts [4].

A priority for Canadian provincial and territorial governments is to ensure equitable access to health services across its communities, particularly for those in hard-to-reach areas. Several challenges exist to delivering equitable cancer care in the northern territories of Canada where 39–52% of the population live in rural communities compared with 19% in Canada as a whole [10]. The Yukon Territory is located in Canada’s northwest and borders Alaska, BC, and the Northwest Territories. It is home to 38,200 residents and 39% are considered rural [10,11]. Most Yukoners live in the southern capital, Whitehorse (29,529). The rest live in remote communities ranging from 53 (Destruction Bay) to 2,202 (Dawson City) in population size [11]. Yukon’s population is growing and ageing [11]. In 2011, the median age was similar to Canada’s (39.4 versus 40.6 years) but the population aged 65 years and over was smaller in Yukon (9.1% and 14.8%) [12]. The Canadian Constitution (1982), recognises three Aboriginal groups in Canada: First Nations, Métis, and Inuit. There are 14 First Nations in Yukon [13], representing nearly a quarter of the population (7,705). There are also 840 Métis and 175 Inuit living in Yukon [14].

In terms of cancer care, some chemotherapies and surgical services are available in Yukon. There is a lack of oncologists in Yukon [15] and Yukoners receive more complex oncology services such as radiotherapy, systemic therapy, and complex surgical procedures in BC and Alberta as they are not available in the territory [16].

Although declining mortality rates in major cancers are well documented nationally [1], it is not clear whether similar progress has been achieved in Canada’s northern territories. The annual Canadian Cancer Statistics publication provides estimates of cancer mortality counts for the present year; however, trends on individual cancers are not provided for Canada’s northern territories in the report, nor are sex-specific trends due to small numbers [1]. A recent study found that cancer is becoming a significant public health problem in Canada’s North as cancer incidence is increasing among Canadian Northern populations [17]. Therefore, there is a need for further examination of cancer mortality patterns in Canada’s North to understand geographic cancer patterns, to identify potential aetiologies, and to inform cancer control priorities. The purpose of this study is to describe cancer mortality in Yukon, to compare with other Canadian regions, and highlight important differences.

## Methods

Yukon cancer deaths between 1 January 1999 and 31 December 2013 were obtained from the Yukon Vital Statistics Registry (YVSR). Data from the YVSR included demographic and cause of death information, as reported on death certificates, for all Yukon residents. Cancer deaths are those for which some form of cancer was listed as the underlying cause of death. Cancer deaths were coded to the *International Statistical Classification of Disease and Related Health Problems, Tenth Revision* [18]. Aggregate summaries of the number of cancer deaths by cancer type were compared with those reported by Statistics Canada’s Canadian Socioeconomic Information Management (CANSIM) database [19], to ensure agreement with the data obtained from YVSR. Data from CANSIM could not be used as the single source for our analysis; data available by age, sex, cancer type, and year in online tools are limited.

We examined Yukon’s cancer mortality rates in relation to national, provincial, and territorial rates. Rates were standardised using direct standardisation to the age structure of the 1991 Canadian population and were calculated per 100,000 person-years. Standard errors used to compute 95% confidence intervals for age-standardised mortality rates (ASMRs) were derived using the Poisson approximation [20]. Comparator data included all-cancer and cancer-specific ASMRs for Canada, and each province and territory; ASMRs were obtained from CANSIM tables [19]. At the time of analysis, the 1991 Canadian population was the standard used for all Canadian health statistics published on the Statistics Canada website, including the cancer mortality information. Therefore, our standard was chosen to be consistent with other data available over the same periods of our study.

Yukon counts of cancer death were pooled annually for all-cancer mortality rates and as cumulative rolling 5-year aggregates for cancer-specific rates, from 1999–2003 to 2009–2013. Because of Yukon’s small population, it was necessary to aggregate data into multi-year periods. At least 20 cancer deaths per 5-year period were required to ensure reliability; only lung cancer met this requirement. The sum of the mid-year populations for the 5 years within each period was used for mortality rate calculations. Similarly, national counts of lung cancer death were pooled as cumulative rolling 5-year aggregates and used as a comparator; counts of cancer death and population data were obtained from CANSIM tables [21,22]. Rates were smoothed using local polynomial smoothing to assess general trends while removing some of the variability resulting from Yukon’s small population from the trend plots. Analysis was conducted using GraphdPad Prism version 7.0a (GraphPad Software, California USA, www.graphad.com).

We also examined Yukon’s cancer mortality relative to sub-provincial comparators. We achieved this by calculating standardised mortality ratios (SMRs) using indirect standardisation [20] relative to the age-specific cancer mortality rates for all of Canada, BC, and three sub-provincial health regions from BC: Interior Health (IH), Northern Health (NH), and Vancouver Coastal Health (VCH). Health authority regions were selected as they shared common health administration boundaries (i.e. NH) and to provide a range of comparisons with regions with similar (i.e. IH and NH) or contrasting rural distributions (i.e. VCH).

SMRs were calculated relative to Canada’s 2008–2012 age-specific cancer mortality rates [22], and the 2009–2013 age-specific rates belonging to BC and its sub-provincial regions. A slightly different period was required for Canada than BC because national data from CANSIM were not available to 2013. Standard errors used to compute 95% confidence intervals for SMRs were derived using the Poisson approximation [20]. BC cancer deaths were obtained from the BC Cancer Registry and aggregated by year, age at death, sex, and health region. Population data were obtained from CANSIM tables [21].

## Results

Yukon and Canada’s distribution of cancer deaths by cancer type were compared for 2008–2012 (Table 1). Yukon and Canada shared similar distributions regarding major cancers. Lung cancer was the leading cause of cancer death for both sexes.

**Table 1.** Percent distribution of cancer deaths by sex, 2008 to 2012.

Yukon (n=164)			Canada [19] (n=189,293)		
Cancer	Cases	Percent	Cancer	Cases	Percent
**Males**					
Lung	50	30%	Lung	52726	28%
Colorectal	22	13%	Colorectal	21376	11%
Prostate	16	10%	Prostate	18727	10%
Stomach	11	7%	Pancreas	9952	5%
Liver	8	5%	Leukaemia	7059	4%
All other cancers	57	35%	All other cancers	79453	42%
Yukon (n=135)			Canada [19] (n=171,369)		
**Females**					
Lung	41	30%	Lung	43395	25%
Breast	24	18%	Breast	24816	14%
Colorectal	15	11%	Colorectal	18421	11%
Pancreas	6	4%	Pancreas	10069	6%
Stomach	6	4%	Ovary	8165	5%
All other cancers	43	32%	All other cancers	66503	39%

For males, the major causes of cancer death in Yukon were proportionally the same as for Canada as a whole: lung, prostate, and colorectal cancer accounted for approximately 50% of all male cancer deaths for these regions. The ordering of the top three causes of male cancer deaths were also identical; lung cancer was the most common, followed by colorectal and prostate. Stomach cancer represented a higher fraction of cancer deaths in Yukon than in Canada as a whole (7% vs 3%) [22]. The fraction of males that died of leukaemia, liver, and pancreatic cancers was approximately the same in Yukon and Canada as a whole (Table 1).

For females, Yukon and Canada showed slightly different proportions of stomach cancer deaths, with 4% of Yukon cancer deaths being stomach cancer (Table 1) compared with 2% for Canada as a whole [22]. Lung, breast, and colorectal cancers accounted for 59% of all cancer deaths in Yukon and 50% in Canada. Pancreatic (4%) and ovarian (4%) cancer deaths were proportionally similar in Yukon to Canada as a whole (6% and 5%, respectively).

**Figure 1.**
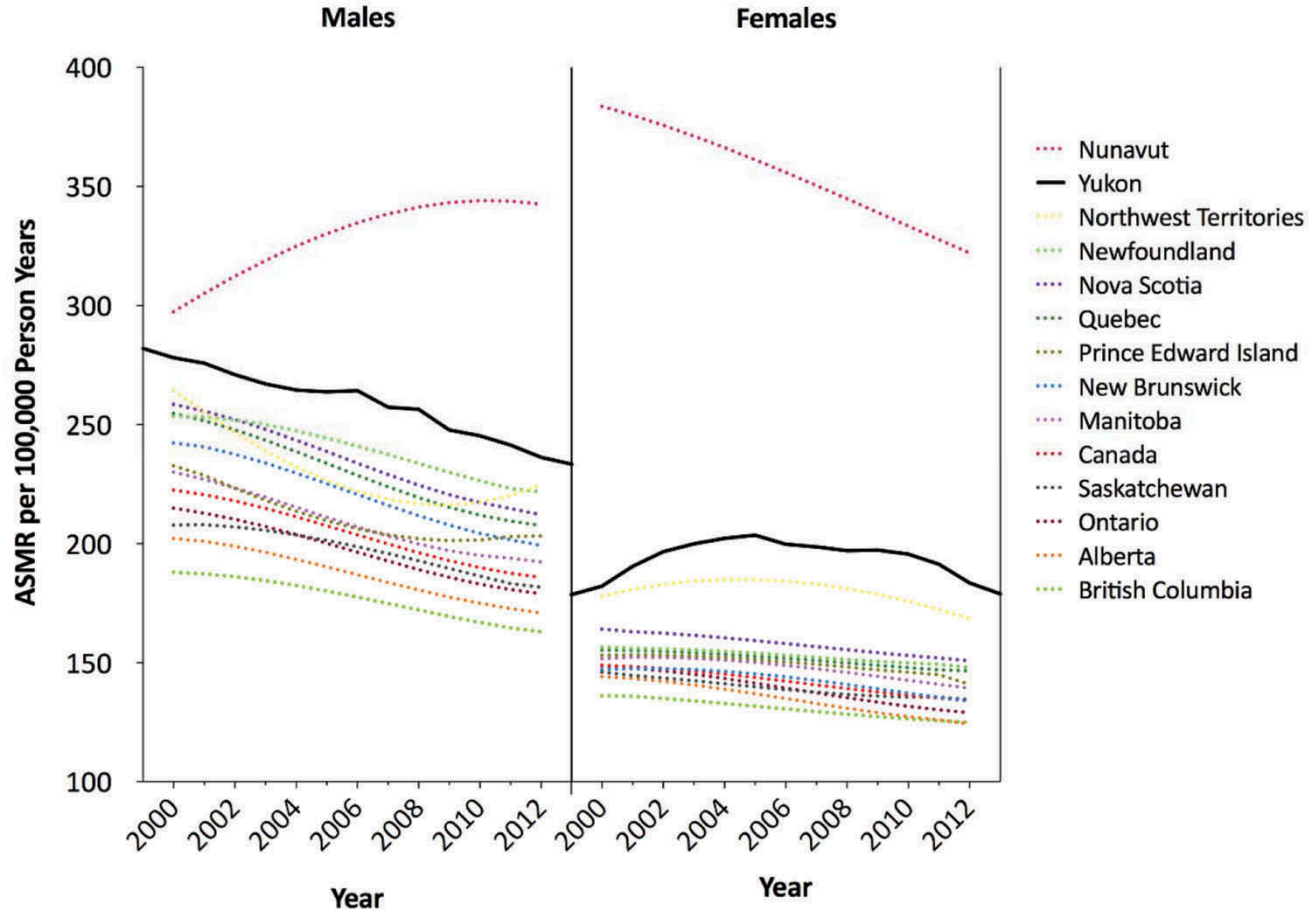
Annual All-cancer ASMRs by sex and region, 1999–2013. National, provincial and territorial ASMRs between 2000 and 2012 were obtained from CANSIM [19].

Yukon annual ASMRs were plotted with national and provincial/territorial annual all-cancer ASMRs (Figure 1). For both males and females, Yukon’s ASMR is elevated above almost all comparator rates for each of the years examined. The trend in all-cancer mortality is declining in the various comparator jurisdictions; Yukon’s all-cancer rates appear to be declining as well for males; however, the trend for females is less clear. The ASMR for all cancers appears to have increased from 1999 to 2005, after which it appears to be declining.

**Figure 2.**
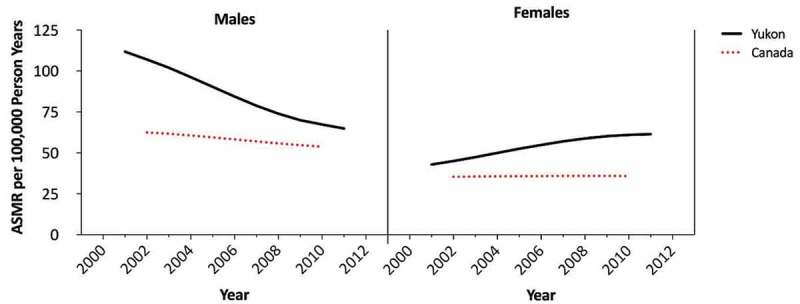
Yukon and Canadian 5-year cumulative rolling lung cancer ASMRs by sex, 1999–2013.

Yukon’s 5-year rolling cumulative lung cancer ASMRs are plotted with national 5-year rolling cumulative lung cancer ASMRs in Figure 2. The male and female ASMRs are elevated above the national rates for each of the years examined. Male lung cancer mortality has been declining since 1999–2003 at a similar, if not greater, rate than the national rate. Yukon’s female trend has been increasing since 1999–2003 at a greater rate than the national rate. It appears to be approaching a plateau in 2009–2013.

Tables 2 and 3 compare the number of observed cancer deaths in Yukon with the number of expected cancer deaths relative to the age-specific cancer mortality rates of a given reference population. Yukon all-cancer and cancer-specific mortality was generally elevated compared with all comparators but most similar to NH. Comparisons drawn against VCH showed the greatest disparities in mortality: prostate (SMR_VCH_=246.3, 95% CI 140.9–351.6), total lung (SMR_VCH_=155.4, 95% CI 138.0–172.7), female breast (SMR_VCH_=169.0 95% CI, 101.4–236.7), and total colorectal (SMR_VCH_=149.3, 95% CI 101.8–196.8) cancers were all significantly elevated in Yukon. In general, female ratios were elevated to a greater extent than male ratios, regardless of the chosen comparator. This was most evident for lung and colorectal cancer mortality. Total stomach cancer mortality was elevated in Yukon relative to all comparators (SMR_BC_=325.4, 95% CI 175.1–475.8, SMR_Canada_=259.5, 95% CI 136.1–382.9, SMR_IH_=350.0, 95% CI 188.3–511.7, SMR_VCH_=299.9, 95% CI 161.3–438.4, SMR_NH_=257.7, 95% CI 138.6–376.6).

**Table 2.** Yukon SMRs relative to age-specific reference rates (BC, BC health authorities, and Canada) by sex and cancer type, 2009–2013. Canadian comparisons are drawn for the 2008–2012 period.

Cancer site		All cancer					Lung					Colorectal				
Region	Sex	O	E	SMR	95% CI		O	E	SMR	95% CI		O	E	SMR	95% CI	
**IH**																
	Male	171	140	122.2*	103.9	140.5	49	37	131.1	94.4	167.8	21	17	124.3	71.1	177.4
	Female	137	108	127.1*	105.9	148.4	42	31	137.0	95.5	178.4	17	11	152.4	79.9	224.8
	Total	308	246	125.3*	111.3	139.3	91	68	134.3*	106.7	161.9	38	28	137.6	93.8	181.3
**VCH**																
	Male	171	114	149.8*	127.3	172.2	49	27	180.3*	129.8	230.8	21	15	139.5	79.8	199.2
	Female	137	86	158.4*	131.9	184.9	42	19	221.2*	154.3	288.1	17	11	158.8	83.3	234.3
	Total	308	198	155.4*	138.0	172.7	91	45	200.1*	159.0	241.2	38	25	149.3*	101.8	196.8
**NH**																
	Male	171	161	106.4	90.5	122.4	49	44	111.1	80.0	142.2	21	19	111.2	63.7	158.8
	Female	137	120	114.0	94.9	133.1	42	38	109.6	76.5	142.8	17	13	126.5	66.4	186.6
	Total	308	280	110.0	97.7	122.3	91	82	110.6	87.9	133.3	38	32	118.0	80.5	155.5
**BC**																
	Male	171	130	131.1*	111.4	150.7	49	33	150.1*	108.1	192.1	21	16	130.6	74.7	186.4
	Female	137	101	135.5*	112.8	158.2	42	26	162.5*	113.3	211.6	17	11	150.3	78.8	221.7
	Total	308	229	134.3*	119.3	149.3	91	58	156.7*	124.5	188.9	38	27	140.5	95.8	185.2
**CA**																
	Male	164	144	113.8	96.3	131.2	50	41	121.1	87.6	154.7	22	16	135.7	79.0	192.4
	Female	135	108	125.2*	104.1	146.3	41	28	144.6*	100.4	188.9	15	10	143.9	71.1	216.7
	Total	299	248	120.6*	106.9	134.3	91	68	133.2*	105.8	160.5	37	26	141.9	96.2	187.7

*Statistically significant p<0.05.

O=Observed, E=Expected, CI=Confidence Intervals, SMR=Standardised Mortality Ratio, IH=Interior Health Authority, VCH=Vancouver Coastal Health, NH=Northern Health Authority, BC=British Columbia, CA=Canada

**Table 3.** Yukon SMRs relative to age-specific reference rates (BC, BC health authorities, and Canada) by sex and cancer type, 2009–2013. Canadian comparisons are drawn for the 2008–2012 period.

Cancer site		Breast					Prostate					Stomach				
Region	Sex	O	E	SMR	95% CI		O	E	SMR	95% CI		O	E	SMR	95% CI	
**IH**																
	Male						21	13	165.3	94.6	236.1	11	3	340.0*	139.1	540.9
	Female	24	16	146.7	88.0	205.4						7	2	356.4	92.4	620.3
	Total											18	5	350.0*	188.3	511.7
**VCH**																
	Male						21	9	246.3*	140.9	351.6	11	4	297.9*	121.9	474.0
	Female	24	14	169.0*	101.4	236.7						7	2	287.5	74.5	500.5
	Total											18	6	299.9*	161.3	438.4
**NH**																
	Male						21	16	127.8	73.1	182.4	11	4	261.7*	107.0	416.3
	Female	24	17	140.0	84.0	196.1						7	3	249.7	64.7	434.7
	Total											18	7	257.7*	138.6	376.7
**BC**																
	Male						21	11	189.8*	108.6	270.9	11	4	299.2*	122.4	476.1
	Female	24	16	146.9	88.1	205.6						7	2	354.5	91.9	617.1
	Total											18	6	325.4*	175.1	475.8
**CA**																
	Male						16	11	146.7	74.8	218.6	11	5	243.9	99.7	388.0
	Female	24	17	138.4	83.0	193.7						6	2	265.4	53.0	477.8
	Total											17	7	259.5*	136.1	382.9

*Statistically significant p<0.05.

O=Observed, E=Expected, CI=Confidence Intervals, SMR=Standardised Mortality Ratio, IH=Interior Health Authority, VCH=Vancouver Coastal Health, NH=Northern Health Authority, BC=British Columbia, CA=Canada

## Discussion

Cancer is an emerging public health issue in Canada’s North. This is the first study to report on cancer mortality in Yukon, a northern and rural region in Canada. We found that all-cancer mortality rates in Yukon were elevated relative to provincial comparators and Canada as a whole. Further, cancer-specific mortality was generally elevated relative to Canadian, BC, and BC sub-provincial comparators. Disparities in mortality were most evident with female lung and stomach cancer, but also with female breast, colorectal, and prostate cancer. We also found rural–urban disparities, evident in all comparisons drawn against VCH, an urban centre in BC. Northern–Southern disparities were evident as Yukon’s cancer mortality was similar to rates from NH, a rural region located in northern BC, but elevated compared with all other regions, including IH, a rural region in southern BC.

Canadian rural–urban differences in cancer mortality appear to be changing over time, notably for lung and colorectal cancer. From 1986 to 1996, all-cancer, lung, breast, and colorectal cancer mortality rates were generally lower among rural Canadians [7]. In a recent Pan-Canadian report, lung, and colorectal cancer mortality rates were elevated among rural Canadians [2]. Rural–urban disparities are multi-faceted and related to differences in modifiable risk factors, access to care, screening, and end-of-life services, and disadvantages in the social determinants of health [2–4,8,9].

Rural–urban disparities differ globally. While cancer mortality is generally higher among rural populations in the US and Australia [7–9], it is generally lower among rural populations in the UK and Western Europe [23]. In Western Europe, this is partly due to elevated smoking rates in urban areas [23]. In the US, rurality and socioeconomic deprivation are key determinants of cancer disparities [8].

Circumpolar regions are internationally unique because of distinct demographics and health challenges [24]. For example, they have large indigenous populations; 23% of the population in Yukon is Aboriginal as opposed to 4% nationally [25]. In terms of health challenges, barriers include widely dispersed populations, harsh environments, limited resources, and cultural and linguistic differences between Aboriginal and non-Aboriginal populations [24,26]. These characteristics, among others, likely contribute to a distinct cancer picture in Circumpolar Canada. Indeed, a study of cancer incidence in circumpolar populations reported substantial disparities with southern counterparts for lung, colorectal, and female breast cancer, particularly among indigenous populations [17]. Northern–Southern differences are related to rapid social, economic, political, and environmental change in Canada’s North [17,24].

We found large disparities in lung cancer mortality, which largely follows smoking prevalence. In 2014, Yukon smoking prevalence was 26%, but has been as high as 36% in the past decade; national estimates were at 18% and as high as 22% in the past decade [27]. Residential radon in Yukon is also among the highest in Canada [28]. Given the long lag time between exposure and developing lung cancer, lung cancer mortality will likely remain elevated in Yukon for decades to come. Yukon’s present smoking prevalence is greater by two-fold that of BC; within BC smoking is lower in southern versus northern regions [27]. This smoking pattern is consistent with the patterns we found in lung cancer mortality, with Yukon rates similar to those in northern but not southern BC.

We also found large regional differences in female cancer mortality, as seen with lung and colorectal cancer. This likely relates to smoking prevalence among females in Yukon (24%) which is elevated compared with Canada (15%) and BC (11%) [27]. The difference in smoking prevalence between Yukon females and other Canadian female populations is greater than their male counterparts [27]. In regards to colorectal cancer, additional elevated risk factors include obesity and alcohol consumption [29]. Although early Canadian studies reported that colorectal cancer mortality was lower among rural populations [7], a recent report found the opposite [2]. In Australia, colorectal cancer mortality is also elevated among both sexes in rural areas, but disparities are greater among females [7]. In addition, rurality and sex were associated with advanced stage colorectal cancer; females were more likely to present with advanced stage [30].

Recently, colorectal cancer screening was introduced in Canada. There are presently 12 provincial/territorial programmes. However, disparities have been reported; colorectal cancer screening rates are lower among lower-income, rural, and Northern Canadian populations [2,3,31]. Colorectal cancer screening participation is 41% among the territories and 55% nationally [31]. A recent study found that jurisdictions with well-established colorectal cancer screening programmes generally have greater colorectal cancer screening participation [31]. The Yukon Government recently implemented a colorectal cancer screening programme, ColonCheck Yukon [32]. The absence of an organised programme in Yukon during the analysis period may be reflected in our findings.

Our finding of elevated rates of stomach cancer mortality may be associated with the elevated prevalence of *Helicobacter pylori* infection in Circumpolar Canada [33]. Previous field studies in Northern Aboriginal communities have reported an elevated prevalence compared with national estimates [33,34]. For example, *H. pylori* prevalence in Old Crow (Yukon), Aklavik and Tuktoyaktuk (Northwest Territories) was reported to be 69%, 61% and 58%, respectively [34]; prevalence among dyspeptic patients across Canada is estimated at 30% [35]. Similar findings have been reported among indigenous communities in Alaska, Greenland, and Russia [33,34]. Pathways for *H. pylori* transmission remain unclear [34]. One study suggested that exposure to mice or mouse droppings, previous consumption of untreated water, and household size may be relevant [34]. To our knowledge, only one community in Yukon (Old Crow) has been assessed for *H. pylori* prevalence (69%) [34]; Yukon-wide prevalence and the prevalence among Yukon First Nations and Non-First Nations is unknown.

Stomach cancer is also associated with smoking, alcohol consumption, and dietary factors, such as high salt and low fibre intake [1]. In Yukon, smoking (26%) and heavy drinking (28%) are elevated versus national smoking (18%) and heavy drinking (18%) estimates [27]. Regarding diet, 37% of Yukoners over the age of 12 self-reported consuming five or more servings of fruits and vegetables per day versus 40% of Canadians [29]. Although salt intake in Yukon is not known, the median intake of Canadian adults exceeds daily recommendations [36]. Further, food insecurity is a concern in Canada’s North and above the Canadian average [17,37]. In Yukon, food insecurity is 12.4% versus 8.3% in Canada as a whole [37].

Rural–urban disparities are also characterised by barriers to accessing treatment, end-of-life services and screening [2–6,38]. For rural Canadians, limited availability of health services and travel time to health centres is a common barrier [2,4–6,38]. For cancer care, this may limit treatment options. In BC, population-based studies have shown lower utilisation of radiotherapy and higher mastectomy rates in Northern BC compared with southern counterparts [5,6]. Similar findings have been reported in Ontario and Canada as a whole [2,4]. Cancer care services are limited in Yukon and the nearest cancer centre is approximately 1,500 km away in Northern BC, which opened in late 2012 at the tail end of our analysis. Further research is needed to identify whether cancer care patterns in Yukon differ from those in other regions, such as Northern BC, and Rural Canada.

There are also disparities in Canadian population-based screening whereby rural, Aboriginal, newly landed immigrants, and low-socioeconomic status (SES) populations are less likely to be screened [2,3,6]. In BC, Olson et al. found that rural breast cancer patients were less likely to utilise screening mammography and more likely to present with advanced disease [6]. In Yukon, 60% of women utilise mammography screening [29]; 78% of women participate nationally [31]. Historically, screening mammography rates were lower among rural versus urban Canadians, but the disparity has decreased over time [3]. Of those who do not access screening, low SES, living in rural and remote areas, newly landed immigrants, and Aboriginal populations are over-represented [3].

This study has several limitations. The descriptive nature of this study only allows us to speculate on potential determinants of regional cancer mortality. Also, we used administrative data from the YVSR, which may vary in cause of death reporting; the YVSR is managed by a registrar, limiting this variation. We verified annual counts of cancer deaths with those reported by Statistics Canada to ensure accurate reporting of Yukon cancer mortality. We did not examine income, education, and ethnicity, important factors related to cancer care and mortality [2–4,8]. Lastly, Yukon exhibits low actual mortality counts, limiting our statistical power and the comparisons we were able to make. To mitigate this, we used 5-year aggregates to draw comparisons.

Cancer is a national public health issue and an emerging concern in Canada’s North [17]. Disparities in modifiable risk factors, cancer care, screening, and end-of-life services exist and rural and Northern Canadian populations are at a disadvantage. Despite this, cancer mortality information is limited for Northern Canadian populations and this gap is a barrier to evidence-based decision-making.

In this study, we found elevated cancer mortality rates in Yukon compared with provincial and national rates, as well as disparities between Yukon rates and those in Southern Canada. Further research and surveillance in Yukon and similar jurisdictions will help reverse the evidence deficit and enable a better understanding of cancer in Circumpolar and Rural Canada. Specifically, more comprehensive research is required to elucidate specific disparities, such as elevated prostate, female breast, female colorectal, and stomach cancer mortality, as well as the determinants of regional cancer mortality and future cancer projections. Additional studies are required to examine rates of subpopulations within Yukon, such as those in very remote communities, Aboriginal Peoples, and those of low SES. Elucidation of geographic and population patterns in cancer mortality would help guide research in cancer aetiology as well as planning cancer prevention and control strategies for Canada’s North.
